# Validity of the FINDRISC as a prediction tool for diabetes in a contemporary Norwegian population: a 10-year follow-up of the HUNT study

**DOI:** 10.1136/bmjdrc-2019-000769

**Published:** 2019-11-28

**Authors:** Anne Jølle, Kristian Midthjell, Jostein Holmen, Sven Magnus Carlsen, Jaakko Tuomilehto, Johan Håkon Bjørngaard, Bjørn Olav Åsvold

**Affiliations:** 1 HUNT Research Center, Department of Public Health and Nursing, NTNU, Levanger, Norway; 2 Department of Endocrinology, St Olavs Hospital, Trondheim University Hospital, Trondheim, Norway; 3 Department of Clinical and Molecular Medicine, NTNU, Trondheim, Norway; 4 Department of Public Health Solutions, National Institute for Health and Welfare, Helsinki, Finland; 5 Department of Public Health, University of Helsinki, Helsinki, Finland; 6 Diabetes Research Group, King Abdulaziz University, Jeddah, Saudi Arabia; 7 Department of Public Health and Nursing, NTNU, Trondheim, Norway; 8 K.G. Jebsen Center for Genetic Epidemiology, Department of Public Health and Nursing, NTNU, Trondheim, Norway

**Keywords:** epidemiology, prediction and prevention

## Abstract

**Objective:**

The Finnish Diabetes Risk Score (FINDRISC) is a recommended tool for type 2 diabetes prediction. There is a lack of studies examining the performance of the current 0–26 point FINDRISC scale. We examined the validity of FINDRISC in a contemporary Norwegian risk environment.

**Research design and methods:**

We followed 47 804 participants without known diabetes and aged ≥20 years in the HUNT3 survey (2006–2008) by linkage to information on glucose-lowering drug dispensing in the Norwegian Prescription Database (2004–2016). We estimated the C-statistic, sensitivity and specificity of FINDRISC as predictor of incident diabetes, as indicated by incident use of glucose-lowering drugs. We estimated the 10-year cumulative diabetes incidence by categories of FINDRISC.

**Results:**

The C-statistic (95% CI) of FINDRISC in predicting future diabetes was 0.77 (0.76 to 0.78). FINDRISC ≥15 (the conventional cut-off value) had a sensitivity of 38% and a specificity of 90%. The 10-year cumulative diabetes incidence (95% CI) was 4.0% (3.8% to 4.2%) in the entire study population, 13.5% (12.5% to 14.5%) for people with FINDRISC ≥15 and 2.8% (2.6% to 3.0%) for people with FINDRISC <15. Thus, FINDRISC ≥15 had a positive predictive value of 13.5% and a negative predictive value of 97.2% for diabetes within the next 10 years. To approach a similar sensitivity as in the study in which FINDRISC was developed, we would have to lower the cut-off value for elevated FINDRISC to ≥11. This would yield a sensitivity of 73%, specificity of 67%, positive predictive value of 7.7% and negative predictive value of 98.5%.

**Conclusions:**

The validity of FINDRISC and the risk of diabetes among people with FINDRISC ≥15 is substantially lower in the contemporary Norwegian population than assumed in official guidelines. To identify ~3/4 of those developing diabetes within the next 10 years, we would have to lower the threshold for elevated FINDRISC to ≥11, which would label ~1/3 of the entire adult population as having an elevated FINDRISC necessitating a glycemia assessment.

**Significance of this study**

**What is already known about this subject?**

- The Finnish Diabetes Risk Score (FINDRISC) is a recommended tool for predicting future diabetes.
- Long-term studies examining the performance of the current 0–26 point FINDRISC scale in contemporary populations are lacking.

**What are the new findings?**

- In a Norwegian population followed up from 2006 to 2008 through 2016, FINDRISC of ≥15 out of 26 had a sensitivity of 38% and a specificity of 90% in predicting future diabetes.
- Among people with FINDRISC ≥15, 13.5% developed diabetes within the next 10 years.
- To identify ~3/4 of those developing diabetes, we would have to lower the threshold for an elevated FINDRISC to ≥11, which would label ~1/3 of the entire adult population as having an elevated FINDRISC.

**How might these results change the focus of research or clinical practice?**

- Clinicians and patients should be aware that a FINDRISC ≥15 carries substantially lower 10-year diabetes risk than previously assumed. To identify 3/4 of people subsequently developing diabetes, we would need to apply a cut-off score for elevated FINDRISC that would label 1/3 of the population as being at elevated risk.

## Introduction

Individuals at high risk of type 2 diabetes may reduce their diabetes risk by 30%–60% through intensive lifestyle intervention,^1–5^ and prediction tools are necessary to identify these high-risk individuals.^6^ The Finnish Diabetes Risk Score (FINDRISC)^7^ is the most recommended risk-screening tool that has proved to be a reliable predictor for future and prevalent undiagnosed diabetes in European and other populations.^8–20^ The FINDRISC was developed in a Finnish study population aged 45–64 years and followed up from 1987 to 1997. People with a score of ≥9 out of 20 had a 13% 10-year risk of type 2 diabetes, and those having a score ≥13 had a 30% 10-year risk.^7^ The FINDRISC has later been revised by adding information on family history of diabetes and an additional age category ≥65 years. A score of ≥15 out of 26 on the revised FINDRISC scale has been proposed, for example, in the Norwegian national guidelines for diabetes, to indicate a >30% 10-year risk of diabetes.^21–23^

Despite its widespread use, there is a lack of large, long-term cohort studies that have examined the risk of future diabetes according to the current 0–26 point FINDRISC scale. Further, the diabetes incidence^24 25^ and prevalence of diabetic risk factors such as overweight and obesity^26 27^ have increased substantially since the 1980s and 1990s, which constitute the time period in which the FINDRISC was developed. In addition, there is a lack of studies examining the performance of the FINDRISC in individuals <45 years,^28^ even though the incidence of type 2 diabetes in younger people has increased.^29 30^ For instance, in the UK, the incidence of diabetes in people <40 years increased more than fivefold from 1991–1995 to 2006–2010.^30^ Moreover, our recent analyses suggested that the ability of the FINDRISC to identify prevalent, but undiagnosed diabetes differed between women and men.^28^ We aimed to examine the validity of the FINDRISC in a contemporary risk environment, and in different groups by age and sex, in a Norwegian population-based cohort followed up from 2006 to 2008 through 2016.

## Methods

### Study population

The HUNT Study (Nord-Trøndelag Health Study) is a population-based study in Nord-Trøndelag county, Norway. It consists of four cross-sectional surveys, HUNT1 (1984–1986), HUNT2 (1995–1997), HUNT3 (2006–2008) and HUNT4 (2017–2019). At each survey, all inhabitants in Nord-Trøndelag aged ≥20 years were invited to participate. The HUNT Study consists of interviews, comprehensive questionnaires, clinical measurements and collection of biological samples, as described in detail elsewhere.^31^ The population of Nord-Trøndelag is fairly representative of Norway, except that the county lacks big cities and that income, education level and proportion of immigrants are slightly lower than the Norwegian average.^31^ This study was performed among participants of the HUNT3 survey, in which 50 802 individuals (54.1% of those invited) participated between October 2006 and June 2008.

### FINDRISC and diabetes in the HUNT3 survey

All participants completed a self-administered questionnaire that included information on previously known diabetes and the FINDRISC items. A standardized clinical examination was performed by trained staff. Weight and height were measured with the participants wearing light clothes without shoes, and body mass index (BMI) was calculated as weight (kg) divided by the squared value of height (m). Waist circumference was measured horizontally at the level of the umbilicus with a non-stretchable band, the participant standing and the arms hanging relaxed. FINDRISC was calculated at the screening site using the following variables and scoring system^17 20 23^: age in years (<45, 0; 45–54, 2; 55–64, 3; and ≥65, 4 points), BMI (kg/m^2^) (<25, 0; 25–30, 1; >30, 3 points), waist circumference (cm) (men: <94, 0; 94–102, 3; >102, 4 points, and women: <80, 0; 80–88, 3; >88, 4 points), physical activity (≥30 min/day, 0; <30 min/day, 2 points), daily consumption of fruits, berries or vegetables (yes, 0; no, 1 point), ever regular use of anti-hypertensive medication (no, 0; yes, 2 points), history of high blood glucose measurement (no, 0; yes, 5 points) and family history of diabetes (no, 0; second-degree but no first-degree relative, 3; first-degree relative, 5 points). Participants without previously known diabetes having FINDRISC ≥15 received information about their elevated risk of developing type 2 diabetes. Non-fasting serum glucose was measured in all participants and analyzed by hexokinase/G-6-PDH methodology (Architect ci8200; Abbott, Clinical Chemistry, Illinois, USA) at Levanger Hospital. Participants without known diabetes who had a non-fasting serum glucose ≥9.0 mmol/L were recommended to consult their general practitioner.

The HUNT3 survey also included information on other clinical and lifestyle characteristics associated with diabetes. The self-administered questionnaire included information on smoking habits and history of cardiovascular diseases. Blood pressure was measured three times at 1 min intervals using an automated blood pressure monitor based on oscillometry (Dinamap 845XT; Critikon, Tampa, Florida, USA). The mean values of the second and third measurements were used in the analyses, and either the second or third measurement alone was used if one of them was missing. Concentrations of total cholesterol, high-density lipoprotein cholesterol, triglycerides and creatinine were measured in non-fasting serum samples.^31^

### Norwegian Prescription Database (NorPD)

We used the unique 11-digit identification number assigned to all Norwegian residents to link the HUNT study data to prospectively recorded information on glucose-lowering medication (ATC classes A10A and A10B) from the Norwegian Prescription Database (NorPD) at the Norwegian Institute of Public Health. NorPD contains information on all drug dispensing to non-institutionalized Norwegian residents since January 2004, and information from 2004 through 2016 was available for the present study. The proportion of filled prescriptions that contained complete ID numbers, enabling inclusion in the database, was 96%–98% in 2004–2005, 98–99% in 2006–2009 and ~100% from 2010 onward. We used incident dispensing of glucose-lowering medication as an indicator for incident diabetes.

### Statistical analysis

Among 50 802 participants in the HUNT3 survey, 47 804 participants were included in the present analysis. Excluded were 2264 people with self-reported diabetes, 151 did not report diabetes but had glucose-lowering medication dispensed between January 2004 and participation in HUNT3, 22 did not report whether or not they had diabetes at HUNT3, 1 formally emigrated out of Norway shortly before participation in HUNT3, 1 could not be linked to NorPD (invalid ID for linkage), and 559 lacked information on one or more FINDRISC items. Participants were followed up from date of participation in HUNT3 until development of diabetes (as indicated by the date of first dispensing of glucose-lowering medication), migration out of Norway, death or December 31, 2016, whichever occurred first.

To assess the validity of the FINDRISC in predicting future development of diabetes, we used receiver operating characteristic (ROC) analysis to estimate the C-statistic (area under the ROC curve, with 95% CI) for the FINDRISC, and to estimate sensitivity and specificity at each possible cut-off value for the FINDRISC, overall and by sex. We estimated the 10-year cumulative incidence of diabetes (with 95% CI) using the Stata stcompet command, taking into account death as a competing risk. We estimated the 10-year diabetes incidence for people with FINDRISC <15 and ≥15 points, corresponding to the current clinical use of the FINDRISC, both in the overall study population and by combinations of sex and age at baseline (<45, 45–54, 55–64, 65–74 and ≥75 years). In the overall population, we also estimated the diabetes incidence in finer FINDRISC categories that have been applied in several previous studies^11 16^; 0–3, 4–6, 7–10, 11–14, 15–19 and 20–26. For illustrative purposes, we additionally plotted both the number of participants and the 10-year diabetes risk for each FINDRISC value. In a separate analysis, we replaced the full FINDRISC model with the concise model excluding information on physical activity and consumption of fruit, berries and vegetables.^7^

In sensitivity analyses, we first excluded metformin use as an indicator of diabetes in women aged <45 years to assess the possible bias introduced by metformin use for other indications than diabetes (eg, polycystic ovary syndrome). Second, we excluded short-term use of glucose-lowering medication (indicated by a time span of <1 year between the first and last dispensing) as an indicator of diabetes in women aged <45 years to assess the possible influence of medically treated gestational diabetes on the estimates. Third, all people with FINDRISC ≥15 at baseline had been informed about their elevated diabetes risk on participation in HUNT3; this information could have influenced the subsequent diabetes incidence both through subsequent lifestyle changes and through increased diagnostic awareness. To assess the impact of this information on diabetes incidence, we estimated diabetes incidence among participants with FINDRISC of 14 and 15 at baseline. These two groups would have relatively similar diabetes risk, but those with FINDRISC of 14 had not been informed about increased diabetes risk. Fourth, all people with FINDRSC ≥15 at baseline had been invited to receive basic lifestyle advice and participate in an oral glucose tolerance test (OGTT), and people without OGTT-defined diabetes were invited to a 2-year diabetes prevention study with repeated lifestyle advice and OGTTs.^32^ Therefore, for people with FINDRISC ≥15, we examined the 10-year diabetes incidence separately among participants and non-participants in the baseline OGTT to assess the extent to which the OGTT and subsequent intervention study might have influenced the incidence estimates. To examine the extent to which diabetes was not captured by the information from NorPD (because diabetes was not treated with drugs or because the participant was institutionalized), we calculated the proportion of people with self-reported diabetes at HUNT3 (self-report of diabetes in HUNT has very high positive predictive value^33^) who had no dispensing of glucose-lowering medication recorded in the NorPD.

Finally, we examined whether the performance of the FINDRISC in our study population could be improved by re-estimating the scores of the individual FINDRISC components. We fit a multivariable Cox proportional-hazards model with all the FINDRISC items and retained categories from the original model, the only exception being that we also included sex as a predictor for diabetes. The inclusion of sex in the model was not based on data-driven variable selection, but on a priori knowledge that male sex was associated with increased diabetes incidence in the study in which the FINDRISC was developed,^7^ and previous cross-sectional analyses in our cohort indicating that inclusion of sex could improve prediction.^28^ We let a beta coefficient of 0.15 (which was the beta coefficient of the weakest predictor in the model) correspond to one point in the scoring system and subsequently assigned points to all categories as recommended.^34^ We calculated the C-statistic of the updated model. We examined its predictive values using the cut-off value that approached the 78% sensitivity achieved in the original FINDRISC study.^7^ The data were analyzed using Stata MP V.15.1 for Windows (StataCorp, College Station, Texas, USA).

## Results

Among 47 804 individuals, 1761 developed diabetes (as indicated by incident dispensing of glucose-lowering medication) during 421 368 person-years of follow-up (median follow-up, 9.2 years; range, 0–10.2 years), corresponding to an incidence rate of 418 per 100 000 person-years. Table 1 shows baseline characteristics of the participants.

**Table 1:** Baseline characteristics of 47 804 participants of the HUNT3 survey (2006–2008), by sex and FINDRISC, given as mean (SD) unless otherwise noted

Characteristics	Women			Men		
	Total (n=26 184)	FINDRISC 0–14 (n=22 972)	FINDRISC 15–26 (n=3212)	Total (n=21 620)	FINDRISC 0–14 (n=19 530)	FINDRISC 15–26 (n=2090)
FINDRISC variables						
Age, years	52.3 (16.2)	50.5 (15.9)	64.8 (12.5)	53.0 (15.6)	51.8 (15.5)	64.2 (11.0)
Waist circumference, cm	89.8 (12.5)	88.2 (11.8)	101.3 (11.0)	97.0 (10.3)	95.9 (9.9)	107.4 (8.6)
BMI, kg/m^2^	26.7 (4.7)	26.1 (4.4)	31.1 (4.6)	27.4 (3.7)	27.0 (3.5)	30.8 (3.4)
Physical activity ≥30 min/day, %	78.1	80.9	57.7	75.2	77.6	51.9
Daily fruit, berries or vegetables, %	70.2	70.7	66.5	50.2	50.5	47.8
Ever treated for hypertension, %	20.1	14.2	61.9	20.4	15.9	61.6
Ever measured high blood glucose, %	5.2	2.5	24.3	2.9	1.1	20.3
First-degree relative with diabetes, %	24.1	17.4	72.4	20.6	15.4	70.0
Second-degree but not first-degree relative with diabetes, %	19.5	20.1	14.9	16.0	16.1	15.0
Other variables						
Non-fasting serum glucose, mmol/L	5.3 (1.0)	5.3 (0.9)	5.8 (1.5)	5.6 (1.3)	5.5 (1.2)	6.1 (1.6)
Total serum cholesterol, mmol/L	5.6 (1.1)	5.5 (1.1)	5.8 (1.1)	5.5 (1.1)	5.5 (1.1)	5.4 (1.1)
Serum HDL cholesterol, mmol/L	1.5 (0.4)	1.5 (0.4)	1.4 (0.3)	1.2 (0.3)	1.2 (0.3)	1.1 (0.3)
Non-fasting serum triglycerides, mmol/L	1.4 (0.8)	1.4 (0.8)	1.8 (0.9)	1.8 (1.1)	1.8 (1.1)	2.1 (1.1)
Serum creatinine, μmol/L	75 (15)	74 (14)	80 (20)	90 (18)	89 (18)	95 (23)
Systolic blood pressure, mm Hg	127 (19)	126 (19)	138 (21)	133 (17)	132 (16)	140 (19)
Diastolic blood pressure, mm Hg	71 (11)	70 (11)	73 (11)	76 (11)	76 (11)	80 (11)
Waist:hip ratio	0.87 (0.07)	0.86 (0.07)	0.92 (0.06)	0.94 (0.06)	0.93 (0.06)	1.00 (0.05)
Current daily smoking, %	19.6	20.2	15.2	15.1	15.3	13.2
Cardiovascular disease,* %	5.7	4.3	15.4	10.4	8.8	26.0

*Self-reported history of myocardial infarction, angina pectoris, heart failure or stroke.

BMI, body mass index; HDL, high-density lipoprotein.

The C-statistic (95% CI) of the FINDRISC in predicting future diabetes was 0.77 (0.76 to 0.78) in the overall study population, 0.78 (0.76 to 0.80) in women and 0.77 (0.75 to 0.78) in men. At the conventional cut-off value of ≥15, the FINDRISC had an overall sensitivity of 38% (44% in women and 34% in men) and an overall specificity of 90% (89% in women and 91% in men) (table 2).

**Table 2:** Sensitivity and specificity of FINDRISC in predicting future diabetes* among 47 804 participants of HUNT3 followed up from 2006 to 2008 through 2016, displayed for each possible cut-off value for the FINDRISC, overall and by sex

Definition of elevated FINDRISC	Overall		Women		Men	
	Sensitivity (%)	Specificity (%)	Sensitivity (%)	Specificity (%)	Sensitivity (%)	Specificity (%)
≥1	99.94	2	99.9	3	100	2
≥2	99.6	5	99.5	5	99.7	6
≥3	99.3	8	99.4	7	99.2	10
≥4	99	13	99	12	99	15
≥5	97	19	98	17	97	22
≥6	96	26	97	24	95	29
≥7	95	33	96	31	93	36
≥8	91	42	93	39	89	45
≥9	86	50	88	48	84	54
≥10	81	59	83	56	78	62
≥11	73	67	76	64	69	71
≥12	65	74	70	71	61	77
≥13	56	80	62	78	52	83
≥14	46	86	52	84	41	88
≥15	38	90	44	89	34	91
≥16	30	93	35	92	25	95
≥17	23	96	28	95	19	97
≥18	17	97	21	97	13	98
≥19	12	98	15	98	9	99
≥20	8	99	11	99	5	99.2
≥21	5	99.5	7	99.4	4	99.5
≥22	3	99.7	4	99.7	2	99.7
≥23	2	99.8	2	99.8	2	99.8
≥24	0.8	99.92	0.9	99.94	0.7	99.9
≥25	0.5	99.96	0.8	99.97	0.3	99.95
26	0.2	99.99	0.4	100	0.1	99.98

*As indicated by dispensing of glucose-lowering medication recorded in the Norwegian Prescription Database.

The 10-year cumulative incidence (95% CI) of diabetes was 4.0% (3.8% to 4.2%) in the entire study population, 13.5% (12.5% to 14.5%) for people with FINDRISC ≥15 and 2.8% (2.6% to 3.0%) for people with FINDRISC <15 (table 3). In terms of predictive values, this means that FINDRISC ≥15 had a positive predictive value of 13.5% and a negative predictive value of 100%−2.8%=97.2% for diabetes within the next 10 years.

**Table 3:** 10-year cumulative incidence of diabetes* among 47 804 participants of the HUNT3 survey according to FINDRISC (<15 vs ≥15) at HUNT3, overall and by categories of sex and age at HUNT3

Sex	Age (years)	FINDRISC	Number of people	Number of diabetes cases	10-year cumulative incidence (%, 95% CI)
Any	Any	Any	47 804	1761	4.0 (3.8 to 4.2)
Any	Any	<15	42 502	1085	2.8 (2.6 to 3.0)
Any	Any	≥15	5302	676	13.5 (12.5 to 14.5)
Women	<45	<15	8753	140	1.8 (1.5 to 2.1)
Women	45–54	<15	5214	88	1.9 (1.5 to 2.3)
Women	55–64	<15	4657	114	2.8 (2.3 to 3.4)
Women	65–74	<15	2616	66	2.9 (2.1 to 3.8)
Women	≥75	<15	1732	37	2.1 (1.5 to 2.9)
Women	<45	≥15	213	29	14.7 (9.9 to 20.4)
Women	45–54	≥15	384	47	14.0 (9.9 to 18.9)
Women	55–64	≥15	981	123	13.4 (11.2 to 15.8)
Women	65–74	≥15	890	88	10.1 (8.2 to 12.2)
Women	≥75	≥15	744	59	8.0 (6.2 to 10.1)
Men	<45	<15	6740	125	2.3 (1.8 to 2.9)
Men	45–54	<15	4407	150	3.8 (3.2 to 4.5)
Men	55–64	<15	4387	206	5.0 (4.3 to 5.7)
Men	65–74	<15	2536	105	4.2 (3.5 to 5.1)
Men	≥75	<15	1460	54	3.7 (2.8 to 4.8)
Men	<45	≥15	74	10	13.5 (6.9 to 22.3)
Men	45–54	≥15	311	61	20.2 (15.8 to 25.0)
Men	55–64	≥15	726	120	17.4 (14.6 to 20.5)
Men	65–74	≥15	595	95	16.8 (13.8 to 20.1)
Men	≥75	≥15	384	44	12.5 (9.0 to 16.7)

*As indicated by dispensing of glucose-lowering medication recorded in the Norwegian Prescription Database.

Among people with FINDRISC <15, the 10-year diabetes incidence was low at ~2%–3% in all age groups in women and 2%–5% across age groups in men. Among women with FINDRISC ≥15, the 10-year incidence was ~14% in age groups <65 years, gradually declining to 8.0% at age ≥75 years. Among men with FINDRISC ≥15, the 10-year incidence was 13.5% at age <45 years, 16%–20% in the age groups 45–74 years and 12.5% at age ≥75 years (table 3).

**Figure 1:**
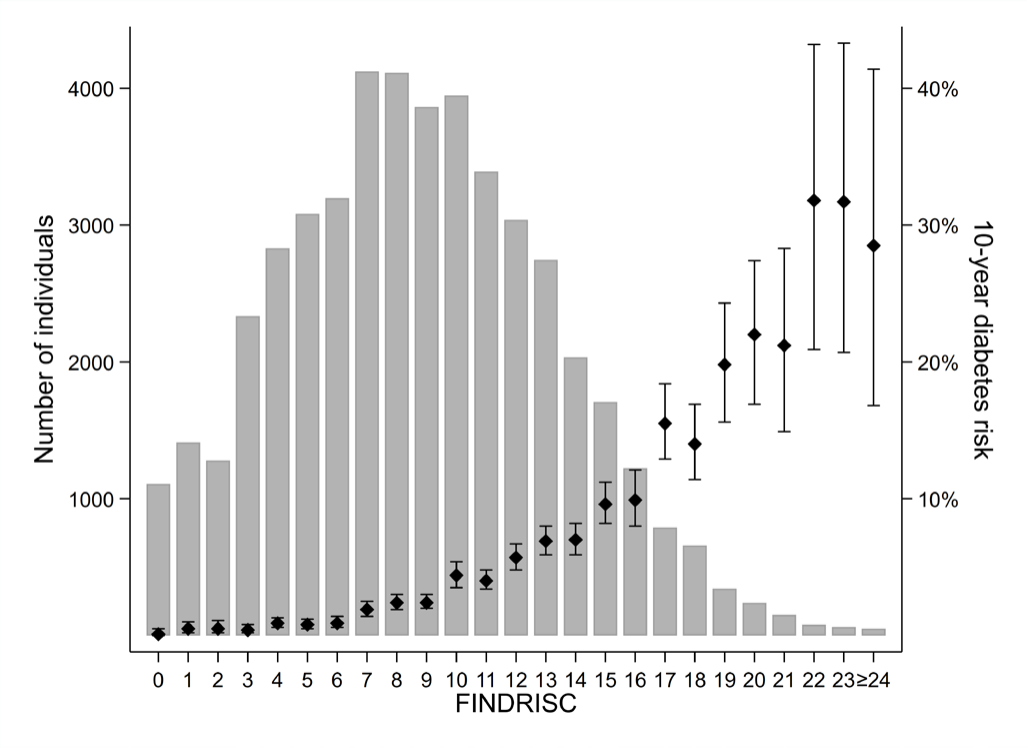
Number of participants and the 10-year diabetes risk (with 95% CI) for each FINDRISC value among 47 804 participants of the HUNT3 survey.

In analysis by finer categories of FINDRISC, the 10-year diabetes incidence was <1% for those with FINDRISC ≤6. The 10-year diabetes incidence gradually increased with increasing FINDRISC, being 2.8% at FINDRISC 7–10, 5.7% at scores 11–14, 12.0% at scores 15–19 and 25.0% at scores ≥20 (table 4). The number of participants and 10-year diabetes incidence for each FINDRISC value are displayed in figure 1.

**Table 4:** 10-year cumulative incidence of diabetes* among 47 804 participants of the HUNT3 survey according to FINDRISC at HUNT3

FINDRISC	Number of people	Number of diabetes cases	10-year cumulative incidence (%, 95% CI)
0–3	6134	23	0.4 (0.3 to 0.6)
4–6	9110	72	0.9 (0.7 to 1.1)
7–10	16 048	389	2.8 (2.5 to 3.1)
11–14	11 210	601	5.7 (5.3 to 6.2)
15–19	4720	536	12.0 (11.0 to 13.1)
20–26	582	140	25.0 (21.3 to 28.7)

*As indicated by dispensing of glucose-lowering medication recorded in the Norwegian Prescription Database.

To approach the 78% sensitivity that was achieved in the study in which the FINDRISC was developed, we would have to lower the cut-off value for elevated FINDRISC to ≥11. This would yield a sensitivity of 73%, specificity of 67% (table 2), positive predictive value of 7.7% and negative predictive value of 98.5%.

The C-statistic of the concise FINDRISC model, which excluded information on physical activity and consumption of fruit, berries and vegetables, was similar to that of the full model. For the concise model, the C-statistic (95% CI) was 0.77 (0.76 to 0.78) in the overall study population, 0.78 (0.76 to 0.79) in women and 0.77 (0.76 to 0.78) in men.

To assess the impact of the information on elevated diabetes risk that was given to all individuals with FINDRISC ≥15 at baseline, we estimated diabetes incidence among participants with FINDRISC on either side of this cut-off, that is, FINDRISC of 14 and 15 at baseline. The 10-year diabetes incidence (95% CI) was 7.0% (5.9% to 8.2%) for people with FINDRISC of 14 and 9.6% (8.2% to 11.2%) for people with FINDRISC of 15. All people with FINDRISC ≥15 were invited to an OGTT shortly after HUNT3, and this OGTT was followed by an invitation to a diabetes prevention study with lifestyle advice among all participants without OGTT-defined diabetes. Among 2692 individuals (with 328 incident diabetes cases) with FINDRISC ≥15 who did not attend the OGTT, the 10-year diabetes incidence (95% CI) was 12.8% (11.5% to 14.2%). Among 2610 attendants (with 348 incident diabetes cases) of the OGTT, the incidence was 14.1% (12.7% to 15.7%). Of the 2610 attendants, 2365 did not have diabetes according to the OGTT performed shortly after HUNT3, and among those 2365 attendants (with 204 incident diabetes cases), the 10-year diabetes incidence was 9.4% (8.1% to 10.8%).

When we excluded treatment with metformin only or treatment of <1-year duration from the diabetes definition among women <45 years of age, the 10-year diabetes incidence in this subgroup was substantially reduced as expected, but these exclusions had little impact on the estimated 10-year incidence in the overall study population (electronic supplementary material (ESM) online supplementary table 1).

10.1136/bmjdrc-2019-000769.supp1 Supplementary data

To examine the extent to which diabetes was not captured by the information from NorPD (due to the diabetes not being treated with medication or because the participant was institutionalized), we calculated the proportion of people with self-reported diabetes at HUNT3 who had no dispensing of glucose-lowering medication. Among 2264 individuals with self-reported diabetes in HUNT3, 427 (18.9%) had no dispensing of glucose-lowering medication between the inception of NorPD in 2004 and the date of participation in HUNT3 between 2006 and 2008, and 207 (9.1%) had no dispensing of glucose-lowering medication between 2004 and 2016. Correspondingly, among 1221 individuals with self-reported diabetes of <10 years’ duration in HUNT3, 276 (22.6%) had no dispensing of glucose-lowering medication between the inception of NorPD and the date of participation in HUNT3, and 113 (9.3%) had no dispensing of glucose-lowering medication between 2004 and 2016.

Finally, we examined if the performance of the FINDRISC could be improved by re-estimating the scores of the individual FINDRISC components (ESM online supplementary table 2). The C-statistic (95% CI) of this updated FINDRISC model in predicting future diabetes was 0.80 (0.79 to 0.81) in the overall study population, 0.81 (0.79 to 0.82) in women and 0.79 (0.77 to 0.80) in men, that is, slightly higher than in the original model. In the updated FINDRISC model, a score of ≥18 out of 41 approached the 78% sensitivity observed in the original FINDRISC study: a score of ≥18 out of 41 had a sensitivity of 75% and specificity of 70% (ESM online supplementary table 3). The positive predictive value for diabetes within the next 10 years was 9.2%, whereas the negative predictive value was 98.5% (ESM online supplementary table 4).

## Discussion

In this population-based prospective cohort study of Norwegian adults without previously known diabetes, the 10-year cumulative incidence of diabetes was 4.0% overall, and 2.8% and 13.5% for those with FINDRISC <15 and ≥15, respectively. The C-statistic for the FINDRISC in predicting future diabetes was 0.77. FINDRISC ≥15, which is the conventional criterion for an elevated FINDRISC necessitating further evaluation or action, had a sensitivity of 38% and a specificity of 90% in predicting future diabetes.

The strengths of this study include the large sample size, which enabled precise estimates of diabetes incidence across all adult age groups. The population-based design indicates that our results are likely representative for the general contemporary adult population in Norway. Nonetheless, the participation rate in the HUNT3 survey was ~50%, and non-participants had lower socioeconomic status, a slightly higher prevalence of known diabetes and higher overall mortality.^35^ Therefore, non-participants may have had a worse glycemic risk profile, which may have led us to underestimate the overall 10-year risk of diabetes in our population.

One limitation is that diabetes was classified based on the dispensing of glucose-lowering medication, which means that the estimates may be influenced by the extent to which diabetes is diagnosed by the general practitioners, and by the decision to use glucose-lowering medication versus lifestyle treatment only. In a recent nationwide Norwegian study, 23.6% of individuals with diabetes did not use glucose-lowering medication.^36^ Similarly, 22.6% of the individuals with self-reported diabetes of <10 years’ duration in HUNT3 did not use any glucose-lowering medication at the time of the survey. If ~23% of the physician-diagnosed incident diabetes cases in our study population were not captured by our prescription data, then the true overall 10-year incidence of physician-diagnosed diabetes may be 5.2% instead of 4.0%, and correspondingly 17.5% instead of 13.5% among people with FINDRISC ≥15. The lack of information about drugs prescribed for institutionalized participants may have led to an underestimation of diabetes incidence at old age since ~10%–15% of those ≥80 years live in institutions.^37^ This may contribute to the decline in the 10-year diabetes incidence at high FINDRISC levels that we observed after 75 years of age.

Our results are likely generalizable to similar and contemporary European populations. However, the validity of the FINDRISC depends on the associations between its individual components and diabetes incidence in the population in which the FINDRISC is applied, and the predictive values of FINDRISC further depend on the diabetes incidence in the population.

In 2012, the Norwegian recommendations on diagnostic testing for diabetes changed from using fasting glucose/OGTT to using HbA1c as the primary diagnostic test for diabetes. Studies have shown inadequate overlap between OGTT-diagnosed diabetes and HbA1c-diagnosed diabetes, and using HbA1c as the sole diagnostic test may have resulted in fewer people being diagnosed with type 2 diabetes among overweight and obese adults.^38–40^ Moreover, our outcome variable included glucose-lowering treatment for all types of diabetes; nonetheless, incident diabetes in adult age is most often type 2 diabetes. Our incidence estimates among women in fertile age should be interpreted with caution, as glucose-lowering medication may be used in the treatment of polycystic ovary syndrome and gestational diabetes. Finally, inviting all people with FINDRISC ≥15 to OGTT at baseline may have resulted in a higher proportion of diabetes cases being diagnosed in this group compared with people with lower FINDRISC.

Lack of comparable, long-term population-based studies using the 0–26 point FINDRISC scale makes it difficult to directly compare our findings with previous studies. Nonetheless, the C-statistic for the FINDRISC in predicting future diabetes was to 0.85 and 0.87 in the original 1987 and 1992 cohorts, compared with 0.77 in our study population, 0.742 in the DETECT-2 study,^41^ and 0.68 for men and 0.81 for women in the DESIR study,^42^ the two latter studies using slightly different versions of the FINDRISC and outcome variables. Our 10-year overall diabetes incidence (4.0%) is similar to the incidence in the Finnish cohort from 1987 in which the FINDRISC was developed (4.1%), both studies using glucose-lowering medication as a proxy for their diabetes incidence. In several official diabetes guidelines and risk assessment forms from national diabetes associations, a FINDRISC of ≥15 out of 26 is assumed to correspond to an approximately 30% 10-year risk of diabetes,^21–23 43–45^ that is, a similar risk as that observed among people with a score of ≥13 out of 20 in the original Finnish study. However, in our cohort, the 10-year diabetes incidence was only 13.5% among those with FINDRISC ≥15 out of 26, that is, similar to the risk observed among people with a score of ≥9 out of 20 in the original Finnish study.

In our study, FINDRISC ≥15 out of 26 had a sensitivity of 38% and a specificity of 90% for predicting future diabetes. In contrast, the criterion originally suggested to indicate elevated FINDRISC, a score of ≥9 out of 20, had a sensitivity of 78% and a specificity of 77% in the original Finnish cohort.^7^ To approach a similar sensitivity, we would have to define FINDRISC of ≥11 out of 26 as an elevated score. This would yield a sensitivity of 73%, but the specificity would drop to 67% (ie, 33% of those not developing diabetes within the next 10 years would have an elevated FINDRISC), and only 8% of people with elevated score would develop medically treated diabetes within 10 years.

In the national diabetes guidelines in Norway, it is suggested that the general practitioners calculate FINDRISC for patients considered at increased risk of type 2 diabetes, and that individuals with score ≥15 are followed up with laboratory assessments and are offered intensive low-threshold multifactorial lifestyle intervention if they have HbA1c or glucose levels indicating intermediate hyperglycemia. The reason to screen is to identify the population who would subsequently develop type 2 diabetes if left untreated, for the purpose of including them in suitable intervention programs.^6^ If the main purpose a diabetes prediction tool is to prioritize people with very high absolute diabetes risk (eg, ~30% or higher 10-year risk) for further follow-up and lifestyle intervention programs, then our results suggest that a score of ≥20 could be an appropriate definition of elevated FINDRISC. However, less than 10% of subsequent diabetes cases would be captured using this cut-off level, so such use of the FINDRISC would have little impact on the population-level diabetes incidence. If, in contrast, the prediction tool is meant to be a sensitive and non-invasive first step in diabetes prediction, one could suggest to lower the definition of elevated FINDRISC to a score of ≥11. This would identify 73% of those who subsequently develop diabetes within the next 10 years, but ~1/3 of the adult population would have to have their HbA1c or glucose levels measured to determine whether they are high-risk individuals in need of lifestyle intervention. On the one hand, this could be regarded as a way to avoid laboratory measurements in 2/3 of the population, if the alternative is to measure HbA1c or glucose as a general population screening without prior clinical risk assessment. On the other hand, labeling 1/3 of the population as high-risk individuals, most of whom will not develop diabetes in the next 10 years, may lead to unnecessary worry. We examined whether this less-than-desirable performance of the FINDRISC could be improved by re-estimating scores better suited to this study population, but observed no substantial improvement in its validity.

In conclusion, our results indicate that the validity of the FINDRISC is lower in a contemporary Norwegian population than in the original Finnish cohorts in which it was developed. The risk of developing diabetes if FINDRISC is ≥15 is substantially lower in the Norwegian population than assumed in official guidelines. Most people who develop diabetes over a 10-year period have FINDRISC <15 and will therefore not be captured through a screening using the FINDRISC. By lowering the threshold for an elevated FINDRISC to ≥11, we would identify ~3/4 of those developing diabetes within the next 10 years, but ~1/3 of the entire adult population would have an elevated FINDRISC necessitating a glycemia assessment.
