# Supplementary material for: Validity of the FINDRISC as a prediction tool for diabetes in a contemporary Norwegian population: a 10-year follow-up of the HUNT study
Source: BMJ Open Diabetes Res Care. 2019 Nov 28;7(1):e000769. doi: 10.1136/bmjdrc-2019-000769 (PMC6887494; doi:10.1136/bmjdrc-2019-000769)
Supplement: Supplementary data [file bmjdrc-2019-000769supp001.pdf]

ESM Table 1. Sensitivity analyses using different criteria for medically treated diabetes among women <45 years at baseline.

Definition of diabetes among women <45 ys at baseline	No. diabetes/total among women <45 ys at baseline		10-year diabetes incidence (%, 95% CI) among women <45 ys at baseline		10-year diabetes incidence (%, 95% CI) in the entire study population	
	FINDRISC <15	FINDRISC ≥15	FINDRISC <15	FINDRISC ≥15	FINDRISC <15	FINDRISC ≥15
Any dispensing of glucose-lowering medication after HUNT3 (as in the main analysis)	140/8753	29/213	1.8 (1.5-2.1)	14.7 (9.9-20.4)	2.8 (2.6-3.0)	13.5 (12.5-13.5)
Any dispensing of glucose-lowering medication after HUNT3 except dispensing of metformin only	55/8753	18/213	0.6 (0.5-0.8)	8.7 (5.3-13.0)	2.6 (2.4-2.8)	13.2 (12.3-14.2)
Any dispensing of glucose-lowering medication after HUNT3 as long as the time from first to last dispensing was ≥1 year	65/8753	21/213	0.7 (0.6-0.9)	10.0 (6.4-14.4)	2.6 (2.4-2.8)	13.3 (12.3-14.3)

ESM Table 2. Updating of FINDRISC by re-estimating the scores of the individual FINDRISC components in the current study population.

Variable	Value	β	95% CI	*P*	Score (β=0.15 corresponds to 1 point)
Age, years	<45	0	Reference		0
	45-54	0.32	0.16-0.47	<0.001	2
	55-64	0.61	0.46-0.76	<0.001	4
	≥65	0.55	0.39-0.71	<0.001	4
BMI, kg/m^2	<25	0	Reference		0
	25-30	0.53	0.32-0.73	<0.001	4
	>30	1.31	1.09-1.53	<0.001	9
Waist circumference, cm	Men <94 Women <80	0	Reference		0
	Men 94-102 Women 80-88	0.35	0.13-0.56	0.002	2
	Men >102 Women >88	1.00	0.78-1.22	<0.001	7
Physical activity ≥30 min/day	Yes	0	Reference		0
	No	0.15	0.05-0.26	0.004	1
Daily consumption of fruits, berries or vegetables	Yes	0	Reference		0
	No	0.16	0.07-0.26	0.001	1
Ever regular use of anti-hypertensive medication	No	0	Reference		0
	Yes	0.49	0.38-0.60	<0.001	3
History of high blood glucose measurement	No	0	Reference		0
	Yes	1.14	1.01-1.28	<0.001	8
Family history of diabetes	No	0	Reference		0
	2nd but no 1st degree relative	0.20	0.06-0.34	0.006	1
	1st degree relative	0.66	0.56-0.76	<0.001	4
Sex	Woman	0	Reference		0
	Man	0.62	0.52-0.73	<0.001	4

ESM Table 3. Sensitivity and specificity of the updated[a] FINDRISC in predicting future diabetes[b] among 47,804 participants of HUNT3 followed up from 2006-08 through 2016, displayed for each possible cut-off value for the updated FINDRISC score, overall and by sex.

Definition of elevated FINDRISC score	Overall		Women		Men	
	Sensitivity (%)	Specificity (%)	Sensitivity (%)	Specificity (%)	Sensitivity (%)	Specificity (%)
≥1	99.94	2	99.9	3	-	-
≥2	99.8	4	99.5	6	-	-
≥3	99.7	6	99.4	11	-	-
≥4	99.5	8	99	15	-	-
≥5	99	12	98	20	100	2
≥6	99	16	98	24	99.6	6
≥7	98	20	96	29	99.3	10
≥8	97	23	95	33	99	12
≥9	97	28	94	37	98	17
≥10	96	32	94	40	97	23
≥11	95	36	93	43	96	28
≥12	93	42	92	48	94	34
≥13	91	47	90	52	92	41
≥14	89	52	88	56	90	47
≥15	86	56	86	59	86	53
≥16	83	61	82	64	83	58
≥17	79	66	79	69	80	62
≥18	75	70	73	72	77	66
≥19	71	74	68	77	73	70
≥20	66	78	62	81	70	74
≥21	61	81	55	85	66	77
≥22	57	84	51	88	61	80
≥23	51	87	45	89	56	84
≥24	46	90	39	92	51	86
≥25	38	93	29	95	45	89
≥26	32	94	24	97	38	92
≥27	28	95	21	97	33	93
≥28	22	97	17	98	26	95
≥29	15	98	12	99	18	97
≥30	13	99	10	99.1	15	98
≥31	11	99	8	99.3	13	98
≥32	8	99.2	6	99.5	10	99
≥33	5	99.5	4	99.7	7	99.3
≥34	4	99.7	2	99.8	6	99.5
≥35	3	99.7	1	99.8	5	99.5
≥36	2	99.8	1	99.94	3	99.7
≥37	1	99.9	0.5	100	2	99.8
≥38	0.8	99.93	-	-	1	99.8
≥39	0.7	99.93	-	-	1	99.9
≥40	0.5	99.97	-	-	0.9	99.93
41	0.1	99.98	-	-	0.2	99.97

[a] by re-estimating the scores of the individual FINDRISC components in the current study population
[b] as indicated by dispensing of glucose-lowering medication recorded in the Norwegian Prescription Database

ESM Table 4. 10-year cumulative incidence of diabetes[a] among 47,804 participants of the HUNT3 Survey according to updated[b] FINDRISC score < vs. ≥18 out of 41 at HUNT3, overall and by categories of sex and age at HUNT3.

Sex	Age (years)	Updated FINDRISC	No. people	No. diabetes	10-year cumulative incidence (%, 95% CI)
Any	Any	Any	47,804	1761	4.0 (3.8-4.2)
Any	Any	<18	32,480	434	1.5 (1.4-1.7)
Any	Any	≥18	15,324	1327	9.2 (8.7-9.7)
Women	<45	<18	7989	88	1.3 (1.0-1.6)
Women	45-54	<18	4183	37	1.0 (0.7-1.4)
Women	55-64	<18	3346	44	1.5 (1.1-2.1)
Women	65-74	<18	1858	27	1.6 (1.1-2.3)
Women	≥75	<18	1210	19	1.6 (1.0-2.4)
Women	<45	≥18	977	81	8.8 (7.0-10.9)
Women	45-54	≥18	1415	98	7.7 (6.1-9.6)
Women	55-64	≥18	2292	193	9.2 (7.9-10.5)
Women	65-74	≥18	1648	127	8.1 (6.7-9.7)
Women	≥75	≥18	1266	77	6.1 (4.9-7.5)
Men	<45	<18	5577	37	0.9 (0.5-1.6)
Men	45-54	<18	3202	53	2.0 (1.4-2.6)
Men	55-64	<18	2623	64	2.7 (2.0-3.4)
Men	65-74	<18	1591	45	3.0 (2.2-4.0)
Men	≥75	<18	901	20	2.2 (1.4-3.3)
Men	<45	≥18	1237	98	8.8 (7.0-10.9)
Men	45-54	≥18	1516	158	11.0 (9.4-12.8)
Men	55-64	≥18	2490	262	11.1 (9.8-12.4)
Men	65-74	≥18	1540	155	10.4 (8.9-12.0)
Men	≥75	≥18	943	78	8.6 (6.8-10.6)

[a] as indicated by dispensing of glucose-lowering medication recorded in the Norwegian Prescription Database
[b] by re-estimating the scores of the individual FINDRISC components in the current study population
